# Health Trajectories of Independent and Dependent Centenarians: A Swedish Nationwide Cohort Study

**DOI:** 10.1093/geroni/igaf050

**Published:** 2025-06-23

**Authors:** Shunsuke Murata, Yuge Zhang, Marcus Ebeling, Katharina Schmidt‑Mende, Karin Modig

**Affiliations:** Unit of Epidemiology, Institute of Environmental Medicine, Karolinska Institutet, Stockholm, Sweden; Unit of Epidemiology, Institute of Environmental Medicine, Karolinska Institutet, Stockholm, Sweden; Unit of Epidemiology, Institute of Environmental Medicine, Karolinska Institutet, Stockholm, Sweden; Laboratory of Population Health, Max Planck Institute for Demographic Research, Rostock, Germany; Division of Family Medicine and Primary Care, Department of Neurobiology, Care Sciences, and Society, Karolinska Institutet, Huddinge, Sweden; Academic Primary Health Care Centre, Stockholm, Sweden; Unit of Epidemiology, Institute of Environmental Medicine, Karolinska Institutet, Stockholm, Sweden

**Keywords:** Exceptional healthy longevity, Health trajectories, Registries

## Abstract

**Background and Objectives:**

Although a large proportion of centenarians depend on assistance, many still live at home, independently or with a little formal long-term care. It is of interest to explore this group further and compare them to dependent centenarians.

**Research Design and Methods:**

This register-based cohort included the entire Swedish centenarian population between 2020 and 2022. Centenarians were classified into two groups: those independent of formal long-term care and those dependent on such care. Disease trajectories were observed in historical data from age 67 and onwards and described for myocardial infarction, stroke, hip fracture, dementia, diabetes, and different cancer diagnoses, as well as hospitalizations and the number of prescribed drugs.

**Results:**

Of the 4,277 centenarians, 36% were independent. Compared with dependent centenarians, independent centenarians had lower incidences of stroke and dementia after age 85 and a lower incidence of hip fracture from age 75. They were less often hospitalized and had lower levels of polypharmacy. In regression analysis, women, stroke, hip fracture, dementia, and more prescribed drugs were associated with an increased risk of being dependent at age 100, while being married was associated with a reduced risk.

**Discussion and Implications:**

The health differences between independent and dependent centenarians appeared mainly after life expectancy was exceeded. After this age, differences in incidences of hip fracture, stroke, and dementia became apparent between the groups. This finding underscores that these diseases affect care needs in very old age and that avoiding them is linked to a more independent life as a centenarian.

**Translational Significance:** This research examined centenarians who were either independent or reliant on formal long-term care, assessing their health and sociodemographic statuses earlier in life. Health disparities between the two groups became noticeable primarily after they surpassed the average life expectancy. Following this age, significant differences in the occurrence of hip fractures, strokes, and dementia emerged. These findings underscore that such conditions significantly affect care needs in extreme old age and that avoiding them is linked to a more independent life as a centenarian.

## Background and Objectives

The number of centenarians—individuals who reach 100 years of age—is increasing due to advancements in medicine and healthcare (United Nations, 2022; Willcox et al., 2010). Although centenarians are often seen as pioneers of longevity, studies have shown that they represent a heterogeneous population in terms of health and functional ability (Evert et al., 2003; Franceschi et al., 2000; Gondo et al., 2006). For instance, the New England Centenarian Study classified centenarians into three phenotypes: survivors, delayers, and escapers, based on their ability to manage 10 age-associated diseases (Evert et al., 2003). Other studies suggest that fully avoiding disease is rare among centenarians, with most experiencing at least one chronic condition (Andersen-Ranberg et al., 2001; Takayama et al., 2007; Vetrano et al., 2021). Nevertheless, despite a large proportion requiring assistance, many centenarians continue to live independently at home or with minimal formal long-term care, and only a few limitations in performing basic activities of daily living (ADL; Andersen-Ranberg et al., 2001; Christensen et al., 2008; Vetrano et al., 2021). Our own research has described the utilization of long-term care services, which included help with basic and instrumental ADL at home and care home residence, and shown that 30% of Swedish centenarians live without, or with only a limited amount of, formal long-term care (Murata et al., 2023). This suggests that many centenarians manage diseases rather well and even escape limitations in basic ADL. Utilization of long-term care services is a measure of the degree of disability and affects well-being, which is why independent living is an important factor in maintaining well-being (Lucas, 2007). It is of interest to explore this group further and to see how they differ from the group of centenarians who are dependent on care. Increased knowledge on how to avoid dependency on long-term care in old age can, therefore, contribute to our understanding of how to extend life expectancy without disability.

Several studies have reported that many diseases, as well as polypharmacy and low socioeconomic status, are associated with poorer functional status or living in a care home among centenarians (Ribeiro et al., 2016; Takayama et al., 2007; von Berenberg et al., 2017). A cross-sectional study reported that strokes and fractures were correlated with higher levels of dependence in ADL (Takayama et al., 2007). Moreover, studies have found that centenarians living in care homes are more likely to suffer from dementia, osteoarthritis, and chronic respiratory disease and to be prescribed more medications compared to centenarians without long-term care or home care (Ribeiro et al., 2016; von Berenberg et al., 2017). However, such cross-sectional designs do not allow comparison of health status earlier in life or trajectories towards long-term care dependency. A few cohort studies have examined care trajectories of centenarians and identified predictors for entering long-term care facilities, for example, dementia and being unmarried (Gellert et al., 2018; Poulain & Herm, 2016). However, the long-term health trajectories of centenarians remain largely unexplored. Understanding these trajectories and which diseases or socioeconomic factors increase the risk of dependency at the age of 100 may contribute to a better understanding of healthy aging among exceptionally long-lived individuals. Therefore, in this study, the aim was to identify centenarians who were independent as well as those dependent on formal long-term care and compare them in terms of sociodemographic characteristics and health trajectories using historical prospective data from earlier periods of their lives up to their 100th birthday. We ask the following questions: At what age do we start to observe differences in health between independent and dependent centenarians? Moreover, for which diseases and health conditions are the differences most evident?

## Research Design and Methods

### Data Sources and Study Population

All individuals who reached 100 years during 2020 and 2022 were identified in the Total Population Register and included in the study. The individuals were identified through the Total Population Register, to which the Social Service Register (Meyer, Sandstrom, et al., 2022), the National Patient Register (NPR), the National Cancer Register, the Prescribed Drug Register, and the Longitudinal Integrated Database for Health Insurance and Labor Market Studies (LISA; Ludvigsson et al., 2019) were linked using the unique Swedish personal identification number. Using historical data, the individuals could be followed from the age of 67. Individuals who migrated between the ages of 67 and 100 (*n* = 147) were excluded, as well as individuals residing in municipalities not reporting to the social service register during the month when individuals turned 100 years old (*n* = 8). This study was approved by the Regional Ethics Committee in Stockholm (reference numbers 2011/136-31/5 and 2020-04753). The board waived the need for patient consent.

### Variable Definitions

Formal long-term care was identified in the Social Service Register (Meyer, Sandstrom, et al., 2022) and consisted of help with daily activities or personal care for centenarians in their own home, or personal care if residing in a care home. Centenarians were categorized into two groups: (i) independent centenarians and (ii) dependent centenarians. Independent centenarians were defined as individuals living at home without any formal long-term care (home care) or using less than 40 hr of formal long-term care per month. Dependent centenarians were defined as individuals living at home using 40 hr or more of formal long-term care per month or living in a care home. The choice of 40 hr was chosen based on the distribution of hours and that this level corresponds with the need for personal care and not only instrumental care (Meyer et al., 2022). We believe that the need for personal care, such as help with showering and getting in and out of bed, marks dependency more strongly than the need for instrumental care, like grocery shopping and preparing meals. In sensitivity analyses, we tested different cutoff values.

Measures of the diseases and health conditions considered were the incidence rate of hospitalization per 1,000 person-years, the cumulative incidence of different diseases, and the proportion of prescribed drug count. To show the proportion of the population that has developed the disease at each age, we chose the cumulative incidence. However, for hospitalizations, they are too common so that almost everyone in the population has experienced hospitalization at some age, and more than once; therefore, the incidence rate is a better measure. The incidence rate was estimated from the ages of 72 to 100. Estimation of cumulative incidence began at the age of 72 as well, while occurrences of the diseases were tracked 5 years earlier, starting at the age of 67. The yearly number of drug prescriptions was identified in the Prescribed Drug Register at each age from 86, since the register has been available from July 2005. The number of different drugs was calculated based on the 3rd level of the Anatomical Therapeutic Chemical code. We classified the number of prescribed drugs into four groups: 0, 1–4, 5–9, and 10 or more drugs, and polypharmacy was defined as having five or more prescribed pharmacological subgroups (Masnoon et al., 2017). Myocardial infarction, stroke, and hip fracture were identified based on the main or secondary diagnosis of hospital admissions (Modig et al., 2017). Dementia and diabetes were identified based on diagnosis of outpatient visit records, in addition to hospitalization records (Ludvigsson et al., 2011; Rizzuto et al., 2018). Colorectal, lung, and breast cancer in women, and prostate cancer in men, were identified based on diagnosis from outpatient visit records, hospitalization records, or the Cancer Register. These diseases were selected based on the hypothesis that they affect dependency in old age and the possibility of measuring them well in the Swedish registers (GBD Ageing Collaborators, 2022; Ludvigsson et al., 2011). Detailed International Classification of Diseases (ICD) codes used in this study to identify respective diseases are shown in Supplementary Table S1.

Being foreign-born or born in Sweden and educational status at the age of 70, as well as marital status at the ages of 70, 80, 90, and 99, were obtained from the LISA register.

### Statistical Analysis

The incidence rate of hospitalization was calculated yearly by dividing the number of hospitalizations by person-years at risk. Cumulative incidence was calculated by dividing the cases at each age by the total centenarian population in the respective age group. To account for different sex compositions between the two centenarian groups, all calculations were sex-standardized, except for breast cancer described for women and prostate cancer described for men. Sex-stratified results are shown in the supplementary results. We additionally estimated incidence rate differences for hospitalization, risk differences for the diseases, and prevalence differences of polypharmacy as comparative measures between independent and dependent centenarians. We applied percentile bootstrap methods with 1,000 resamples to calculate the 95% confidence interval (95% CI).

To identify which diseases and indicators were associated with being dependent at age 100, we estimated the risk ratios of some candidate variables using modified Poisson regression (Naimi & Whitcomb, 2020). The following variables were included in the model: prescribed drug count, specific diseases at the age of 100 where we observed differences, marital status at the age of 99, and sex. All data were analyzed using R, version 4.3.1 (R Core Team, 2024).

## Results

A total of 4,277 individuals turned 100 years old between 2020 and 2022 and were included in the study. Most centenarians were women (81%), and 64% (*n* = 2,726) of them were dependent. Among the dependent centenarians, 28% (*n* = 769) lived at home receiving 40 or more hours of home care per month, while the remaining 72% (*n* = 1,957) resided in care homes. Among the 36% (*n* = 1,551) independent centenarians living at home, 56% (*n* = 867) did not use any home care, and the remaining 44% (*n* = 684) used less than 40 hr per month.

Table 1 presents the demographic characteristics of independent and dependent centenarians. Predominantly, individuals in both groups were born in Sweden. The majority of centenarians had experienced the loss of their spouse, with the prevalence of widows increasing progressively with advancing age. The independent group had a higher share of men than the dependent group (25% vs 16%). Furthermore, the proportion of married individuals at the age of 100 was higher among independent than dependent centenarians (8% vs 3%).

**Table 1.** Characteristics of Independent and Dependent Centenarians

Characteristic	Independent centenarians	Dependent centenarians	Total
	*n* = 1,551	*n* = 2,726	*n* = 4,277
Sex			
Women	1,165 (75.1%)	2,279 (83.6%)	3,444 (80.5%)
Men	386 (24.9%)	447 (16.4%)	833 (19.5%)
Birth at Sweden	1,438 (92.7%)	2,557 (93.8%)	3,995 (93.4%)
Birth year			
1920	503 (32.4%)	962 (35.3%)	1,465 (34.3%)
1921	525 (33.8%)	901 (33.1%)	1,426 (33.3%)
1922	523 (33.7%)	863 (31.7%)	1,386 (32.4%)
Education age 70			
Compulsory	919 (60.9%)	1,572 (58.5%)	2,491 (59.3%)
Upper secondary	420 (27.8%)	793 (29.5%)	1,213 (28.9%)
Undergraduate level	171 (11.3%)	324 (12.0%)	495 (11.8%)
Marital status at age 70			
Never married	80 (5.2%)	155 (5.7%)	235 (5.5%)
Married	1,034 (67.1%)	1,742 (63.9%)	2,776 (65.1%)
Divorced/separated	90 (5.8%)	207 (7.6%)	297 (7.0%)
Widower	337 (21.9%)	622 (22.8%)	959 (22.5%)
Marital status at age 80			
Never married	78 (5.1%)	151 (5.5%)	229 (5.4%)
Married	710 (46.0%)	1,203 (44.1%)	1,913 (44.8%)
Divorced/separated	96 (6.2%)	209 (7.7%)	305 (7.1%)
Widower	659 (42.7%)	1,163 (42.7%)	1,822 (42.7%)
Marital status at age 90			
Never married	78 (5.0%)	151 (5.5%)	229 (5.4%)
Married	340 (21.9%)	483 (17.7%)	823 (19.2%)
Divorced/separated	97 (6.3%)	206 (7.6%)	303 (7.1%)
Widower	1,036 (66.8%)	1,886 (69.2%)	2,922 (68.3%)
Marital status at age 99			
Never married	78 (5.1%)	150 (5.5%)	228 (5.3%)
Married	125 (8.1%)	84 (3.1%)	209 (4.9%)
Divorced/separated	95 (6.2%)	208 (7.6%)	303 (7.1%)
Widowed	1,244 (80.7%)	2,284 (83.8%)	3,528 (82.7%)
Long-term care status			
No care	867 (55.9%)	NA	867 (20.3%)
Home care <40 hr	684 (44.1%)	NA	684 (16.0%)
Home care over 40 hr	NA	769 (28.2%)	769 (18.0%)
Care home	NA	1,957 (71.8%)	1,957 (45.8%)

*Notes*: NA = not applicable. Data were expressed with *n* (%). Independent group had 10, 8, and 9 missing cases at marital status at ages 70, 80, and 99, respectively. 78 missing cases for education existed.

**Figure 1.**
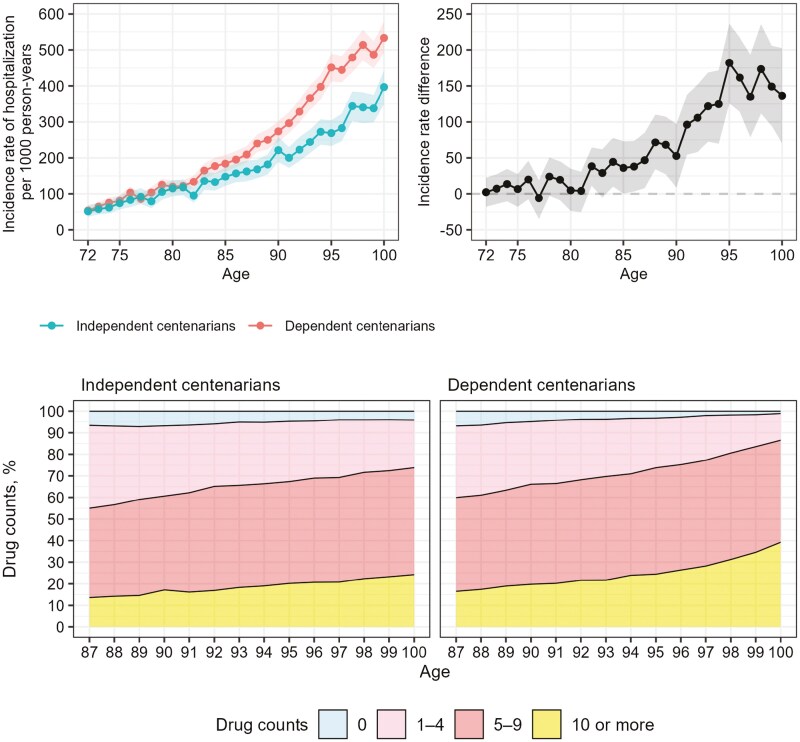
The sex-standardized incidence rates of hospitalization and proportions of drug prescription counts for the two groups of centenarians. Sex-standardized incidence rate of hospitalization from the age of 72 and onwards (upper left panel), incidence rate difference (upper right panel) for independent and dependent centenarians, and the proportion of drugs from the age of 87 (lower panels), respectively. Shaded area represents the 95% confidence interval.

Figure 1 illustrates the sex-standardized incidence rates of hospitalization and proportions of drug prescription counts (0, 1–4, 5–9, and 10 or more) for the two groups of centenarians. The incidence rate of hospitalization increased with age for both independent and dependent centenarians, but the increase was more rapid for dependent centenarians. The differences began to appear after the age of 80 and continued until the age of 95, at which point the increase decelerated. Information on the counts of pharmacological subgroups was available from the age of 87. From this age, a difference in the number of drugs prescribed was observed between independent and dependent centenarians, with a higher proportion of dependent centenarians having 5–9 prescribed drugs. Dependent centenarians consistently demonstrated a higher prevalence of polypharmacy compared to independent centenarians. The detailed results of these differences are presented in Supplementary Figure S1. Stratified analysis by sex yielded results consistent with the sex-standardized findings concerning hospitalization and drug prescription patterns, as depicted in Supplementary Figures S2 and S3.

**Figure 2.**
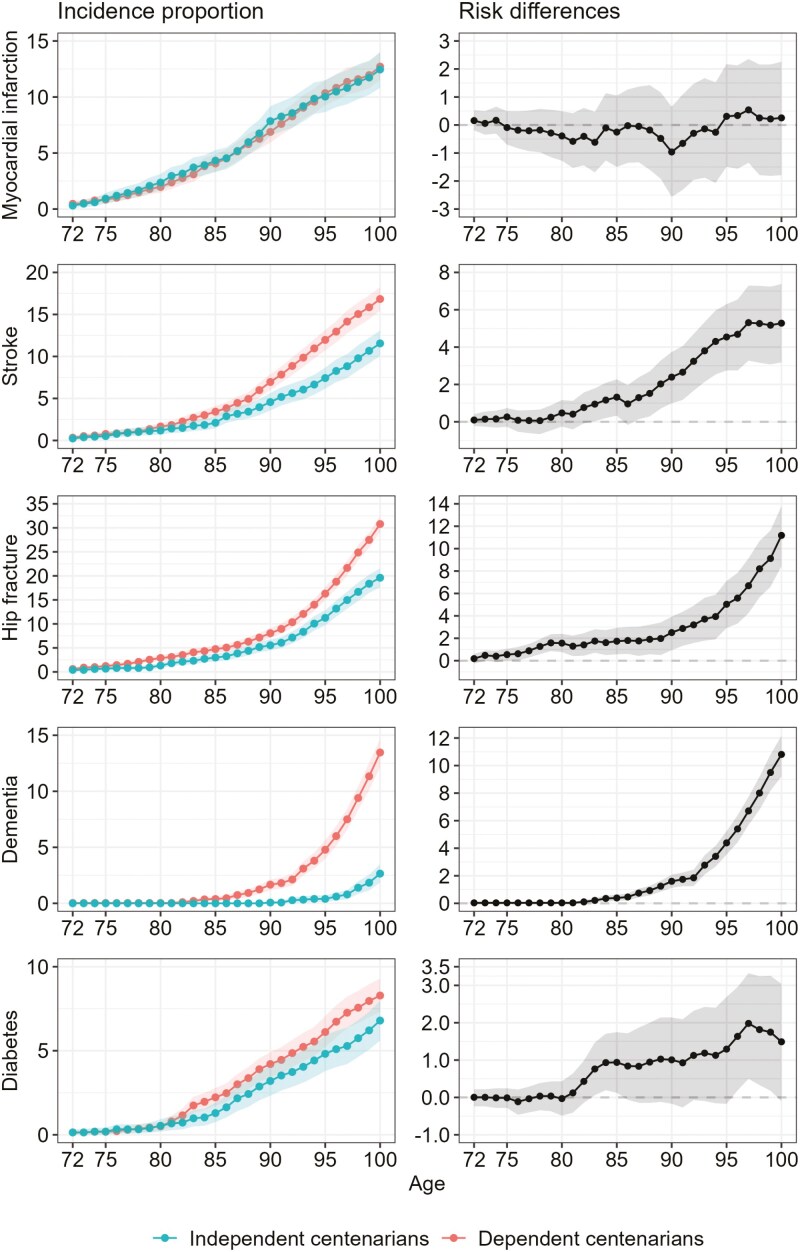
The sex-standardized incidence proportions of different diseases and the respective risk differences between the two groups of centenarians. Sex-standardized cumulative incidence of different diseases from the age of 72 and onwards (left panels) and absolute risk difference (right panels) for independent and dependent centenarians, respectively. Shaded area represents the 95% confidence interval.

**Figure 3.**
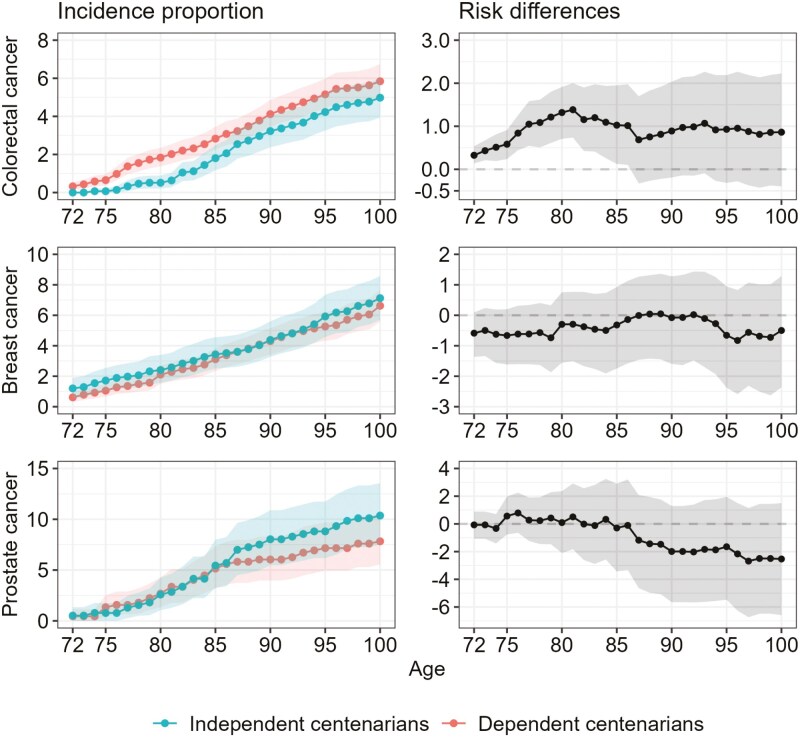
The sex-standardized incidence proportions of different cancers and the respective risk differences between the two groups of centenarians. Sex-standardized cumulative incidence of colorectal-, breast- (women only), and prostate (men only) cancer from the age of 72 and onwards (left panels) and absolute risk difference (right panels) for independent and dependent centenarians, respectively. Shaded area represents the 95% confidence interval.

Figures 2 and 3 depict the sex-standardized incidence proportions of different diseases as well as the respective risk differences between independent and dependent centenarians. The rise in cumulative incidence with age was the same for myocardial infarction in both groups, and the cumulative incidence among both groups was similar. On the contrary, the cumulative incidence for stroke, hip fracture, and dementia in dependent centenarians was higher compared to independent centenarians. It was also higher for diabetes, but the difference was not statistically significant. The onset of stroke and dementia shows a distinct divergence around the age of 85, whereas the distinction occurs somewhat earlier for hip fracture. The cumulative incidence of stroke increased gradually from the age of 72 in both groups. It was higher in dependent centenarians than in independent centenarians, and the difference at the age of 100 was 5%. The exponential rise in dementia after the age of 85 was observed only in dependent centenarians. Only 3% of independent centenarians experienced dementia at the age of 100. The difference among the groups at the age of 100 was 11%. Notably, hip fracture exhibits the highest cumulative incidence among all conditions in both cohorts, with rates of 31% among dependent centenarians and 19% among independent centenarians. The incidence pattern for specific cancers varies. For colorectal cancer, the cumulative incidence rises more steeply up to the age of 80 among dependent centenarians, after which both groups show a similar age-related increase. No disparity was observed for breast cancer, while for prostate cancer, no differences were noted until the age of 85, beyond which independent centenarians displayed a somewhat steeper increase in cumulative incidence compared to dependent centenarians. Lung cancer was almost nonexistent among centenarians (0.2%). Sex-specific cumulative incidence results are detailed in Supplementary Figure S4.

**Figure 4.**
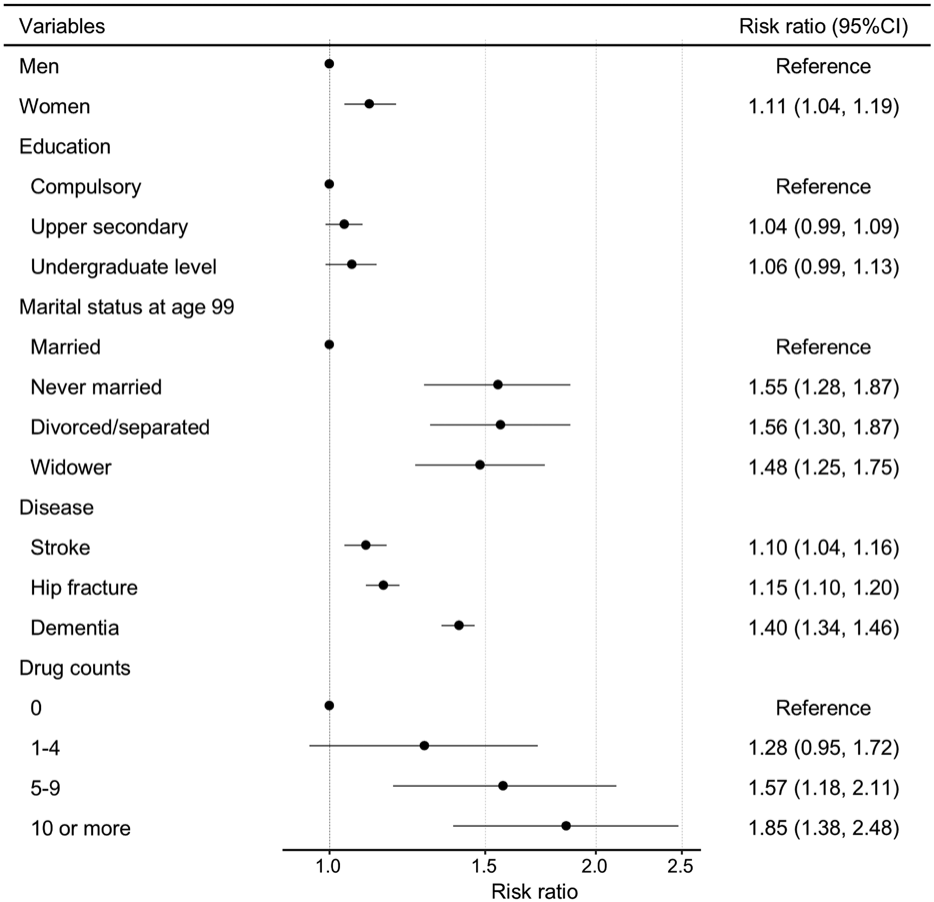
Risk ratios and (95% confidence intervals [CI]) for variables associated with being a dependent centenarian estimated with modified Poisson regression, all estimates mutually adjusted.

Figure 4 presents the result from the regression analysis, estimating the association between different variables and being dependent as a centenarian. Our findings indicate that almost all included variables were associated with an elevated risk of becoming a dependent centenarian except for education. Being a woman slightly increased the risk, with a risk ratio of 1.11, when considering all other variables in the model. Dementia and polypharmacy showed a higher risk for dependency among centenarians, whereas being married was associated with a decreased risk. Compared to being married, individuals who were never married, divorced, or widowed had approximately 1.5 times higher risk of dependency at age 100 [risk ratio and the 95% CI, never married, 1.55 (1.28, 1.87); divorced/separated, 1.56 (1.30, 1.87); widower, 1.48 (1.25, 1.75)]. Centenarians with dementia had a 1.40 (95% CI = 1.34, 1.46) times higher risk than those without dementia. Stroke and hip fracture increased the risk, though to a lesser extent compared to dementia. The risk of dependency among centenarians increased progressively with higher counts of prescribed drugs [risk ratio and the 95% CI, drug counts 1–4, 1.28 (0.95, 1.72); drug counts 5–9, 1.57 (1.18, 2.11); 10 or more, 1.85 (1.38, 2.48)].

## Discussion and Implications

This study identified centenarians who were either independent or dependent on formal long-term care and compared their health and sociodemographic status earlier in life. The findings revealed that independent centenarians had lower incidence rates of hospitalization, lower levels of polypharmacy, and a less steep increase in the cumulative incidence of hip fractures, stroke, and dementia than dependent centenarians. Being a woman, experiencing stroke, hip fracture, or dementia, as well as having been prescribed many drugs, were associated with an increased risk of dependency at the age of 100, while being married decreased the risk. These differences in health status were evident up to two decades before reaching the age of 100, though they became more pronounced primarily after surpassing the average life expectancy. This study further highlights differences within the centenarian population itself, showing that approximately one-third of centenarians delay or even avoid the onset of diseases to a greater extent than the others.

Stroke and hip fracture directly affect physical functioning, often resulting in dependency on long-term care (Takayama et al., 2007). In line with this, our study showed a lower incidence of stroke among independent centenarians compared to dependent centenarians. Although the differences were clear between the two groups, some independent centenarians still experienced a stroke or a hip fracture. This shows that it is possible to maintain or improve the function after these diseases and that rehabilitation and recovery, even at an advanced age, can be effective. Furthermore, we found that dementia incidence was lower in independent centenarians, which is in line with previous studies. Both longitudinal and cross-sectional studies have reported how dementia is associated with long-term care utilization in centenarians (Gellert et al., 2018; von Berenberg et al., 2017). Our study showed how the cumulative incidence of dementia increased exponentially in dependent centenarians after the age of 85, while no large increase was observed in independent centenarians. The regression analyses also showed that dementia had a stronger association with dependence than stroke and hip fracture.

Independent centenarians also experienced fewer hospitalizations and were prescribed a lower number of drugs in the decade leading up to age 100 than dependent centenarians. This is in line with findings that centenarians residing in care homes are prescribed more drugs than centenarians living at home, but the other studies did not find a difference in hospitalization between the two groups (Ribeiro et al., 2016; von Berenberg et al., 2017). It is not surprising that both drug use and hospitalization are related to dependence because they likely reflect higher levels of morbidity and frailty, but it is worth noting that this is still the case at a high age of 100, where only very few individuals are still alive.

Being married at the age of 100 was associated with lower degrees of long-term care utilization, in line with previous findings (Poulain & Herm, 2016). It is likely that married centenarians benefit from informal care or support from their partner, which in turn reduces the need for formal long-term care. Another possible explanation can be loneliness. Widowhood has been associated with depression, loneliness, and physical illness (Stroebe et al., 2007), which could increase the need for long-term care. The role of living arrangement and social support networks for healthy aging and how these might influence aging trajectories is an interesting aspect to follow up in future studies.

Our study has the strength of encompassing the entire centenarian population in Sweden, thereby ensuring a comprehensive representation. Furthermore, our study benefits from a long follow-up without any loss to follow-up. This aspect is particularly significant as sampling cohorts frequently encounter issues such as dropouts and a tendency to include only healthier individuals. Such limitations can impede the ability to compare independent and dependent centenarians effectively. However, some limitations should be considered when interpreting our results. First, dementia was measured by a diagnosis code in the NPR. This has been shown to have a high positive predictive value but lower sensitivity (Rizzuto et al., 2018), resulting in an underestimation of the true prevalence of some diseases, shown by a lower incidence than that of previous studies in centenarians (Leung et al., 2023). Even though underestimation affects all individuals in the same way, it likely underestimates dementia somewhat more in the dependent group, where dementia may be more prevalent. Second, it should be noted that our results depend on the definition of being dependent as having formal long-term care for 40 hr or more per month at home or residing in a care home. We conducted sensitivity analyses by redefining dependency as having personal care rather than instrumental care, which yielded similar results. However, we were unable to account for the fact that many centenarians likely receive informal care. As a proxy, we considered marital status, though this is an imperfect measure that does not capture assistance provided by children. Nonetheless, we believe that many—if not most—centenarians receive some form of informal care, which is likely present in both independent and dependent groups. While the dependent group would remain unchanged, the independent group may include individuals with varying levels of informal support, meaning that some may be less independent than others. Had we been able to identify centenarians who are truly independent, including from informal care, it is likely that the observed health differences between the independent and dependent groups would have been even greater. However, our main finding—observable differences in health outcomes several decades before reaching the age of 100—would remain unchanged. Third, healthy survival effects might affect the estimated risk ratio (Digitale et al., 2023). However, we reduced this bias by including variables such as socioeconomic status and health indicators, that is, number of drugs, in the regression model (Banack et al., 2019). Finally, we did not measure some burdensome diseases and disease severity. Some major burdensome diseases, such as COPD, heart failure, and hypertension, are still not validated in the Swedish register (GBD Ageing Collaborators, 2022). Additionally, stratifying the included diseases by their severity could have provided more insights into how they affect care dependency.

In conclusion, centenarians who were independent of, or only used little, formal long-term care were more likely to have avoided stroke, dementia, and hip fracture earlier in life, compared to centenarians who were dependent on long-term care. No apparent differences were observed for myocardial infarction, diabetes, and cancer. Independent centenarians also experienced fewer hospitalizations, had lower levels of polypharmacy, and were more likely to cohabit than their dependent counterparts. Notably, differences in disease incidence between independent and dependent centenarians became apparent primarily after exceeding average life expectancy but were already visible from around the age of 80. These findings highlight the profound impact of conditions such as stroke, hip fracture, and dementia on care needs at the highest ages, emphasizing that health status much earlier in life not only influences survival but also significantly shapes the quality of life at exceptional ages.

## Supplementary Material

igaf050_suppl_Supplementary_Materials

## Data Availability

The General Data Protection Regulation (GDPR) in Sweden does not permit sharing the data used in this study publicly. This study was not preregistered.
