# Supplementary material for: Health Trajectories of Independent and Dependent Centenarians: A Swedish Nationwide Cohort Study
Source: Innov Aging. 2025 Jun 23;9(6):igaf050. doi: 10.1093/geroni/igaf050 (PMC12289545; doi:10.1093/geroni/igaf050)
Supplement: igaf050_suppl_Supplementary_Materials [file igaf050_suppl_supplementary_materials.docx]

***Innovation in Aging* Supplementary Material: Murata, Zhang, Ebeling, Schmidt-Mende, & Modig. Health Trajectories of Independent and Dependent Centenarians: A Swedish Nationwide Cohort Study.**

**Supplementary Table 1. International Classification of Diseases codes used in this study.**

| Disease | ICD-7 | ICD-9 | ICD-10 |
| --- | --- | --- | --- |
| Myocardial infarction |  | 410 | I21, I22 |
| Stroke |  | 430-432, 434 | I60-I64 |
| Hip fractures |  | 820 | S720-722 |
| Dementia |  | 290, 294, 331 | F00-F03, F051, G30 |
| Diabetes |  | 250 | E11, E14 |
| Colorectal cancer | 153, 154.0 | 153, 154.0, 154.1 | C18-C20 |
| Lung cancer | 162 | 162 | C33-C34 |
| Breast cancer | 170 | 174 | C50 |
| Prostate cancer | 177 | 185.9 | C61 |

**
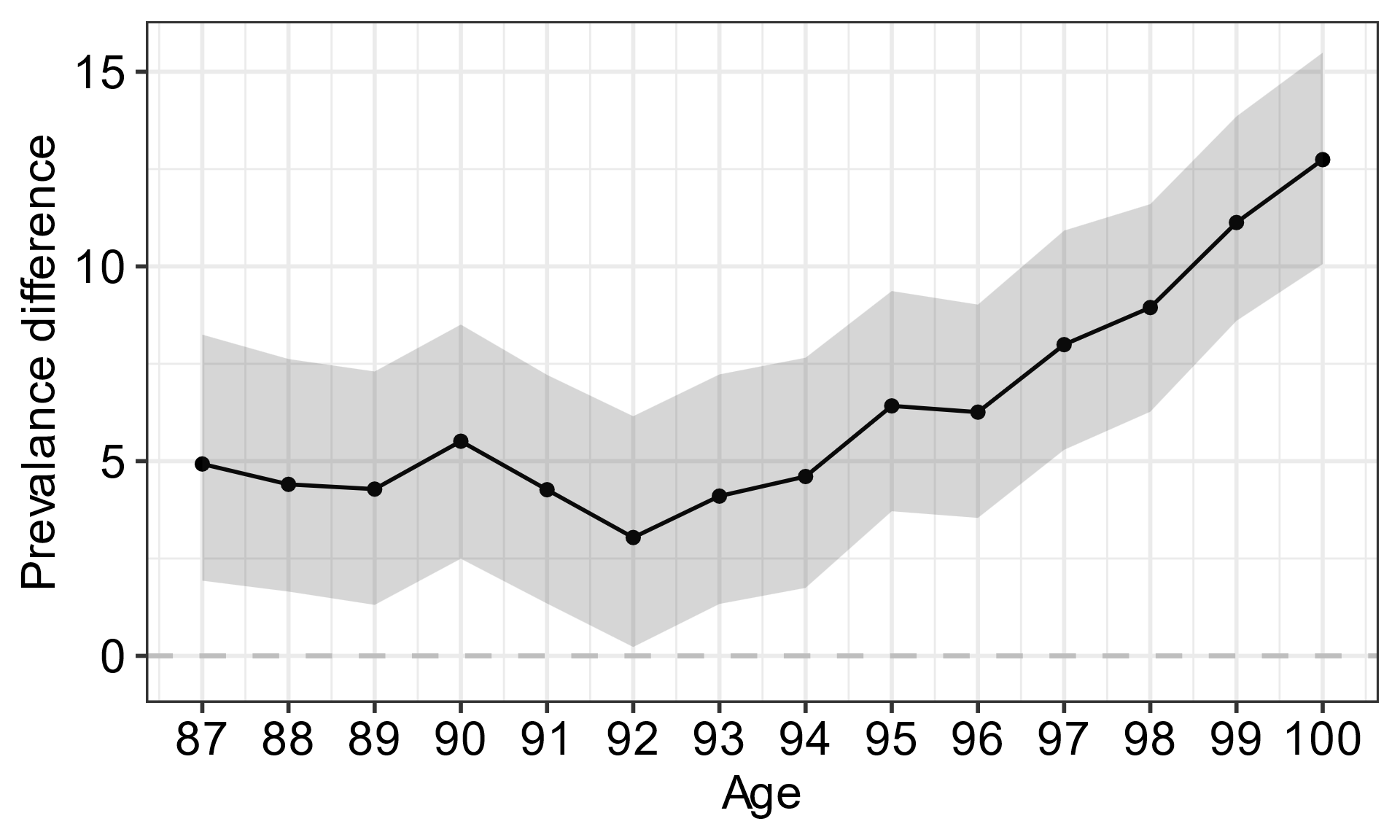
Supplementary Figure 1. Sex-standardized prevalence difference of polypharmacy between independent and dependent centenarians.**

Note. The shaded area represents the 95% confidence interval.

**
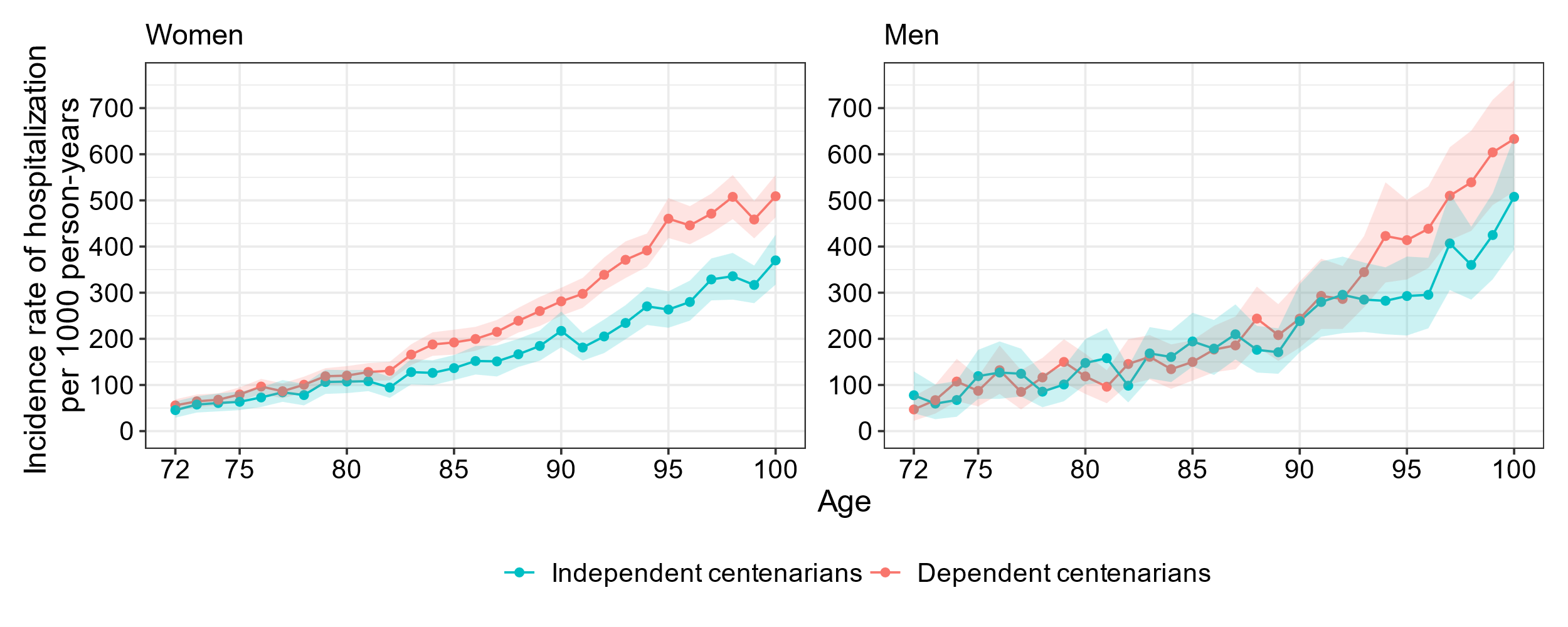
**

**Supplementary Figure 2. Sex stratified incidence rate of hospitalization from the age of 72 and for independent and dependent centenarians, respectively.**

Note. Shaded area represents the 95% confidence interval.

**
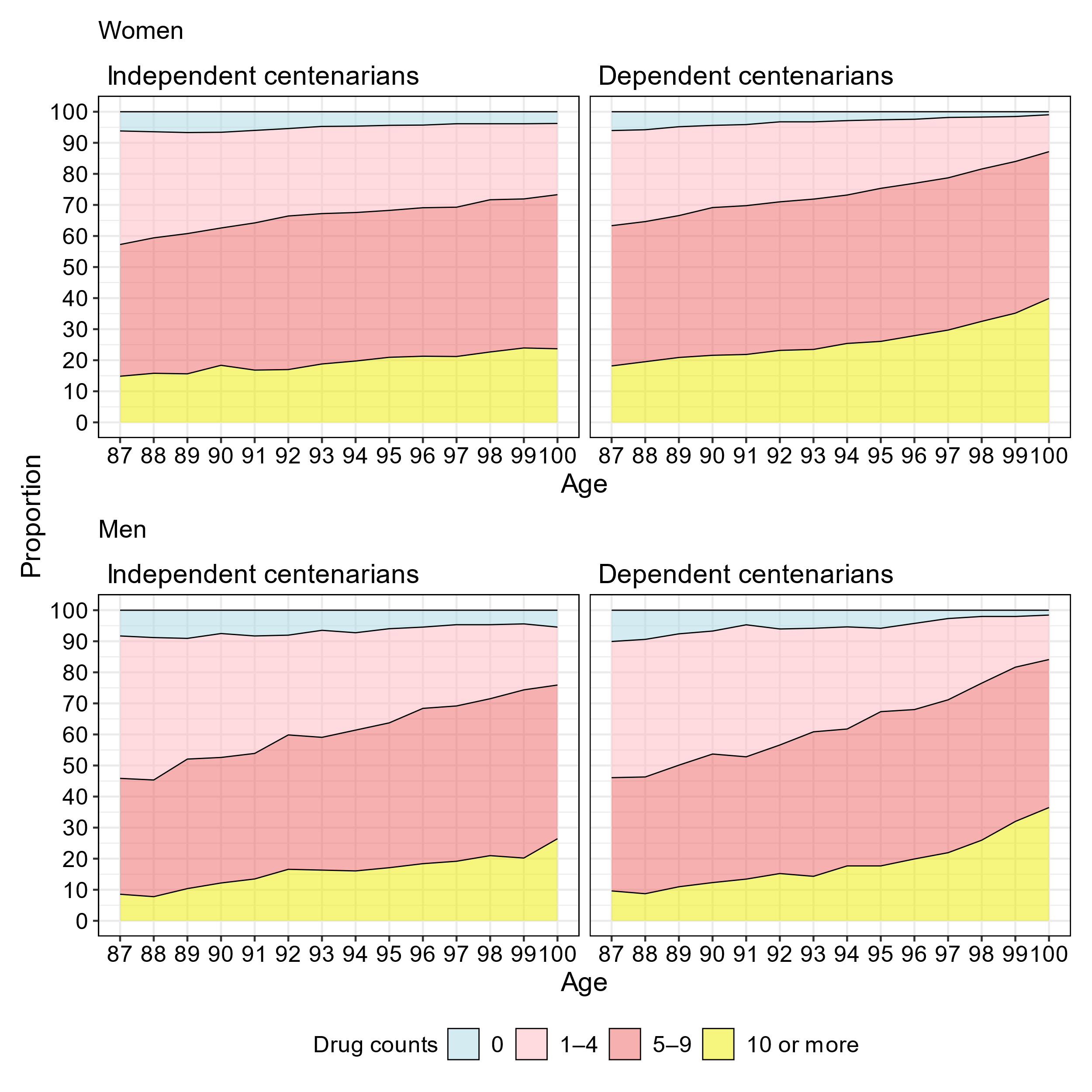
** **Supplementary Figure 3. Sex stratified proportion of drug counts from the age of 87 and onwards for independent and dependent centenarians.**

**
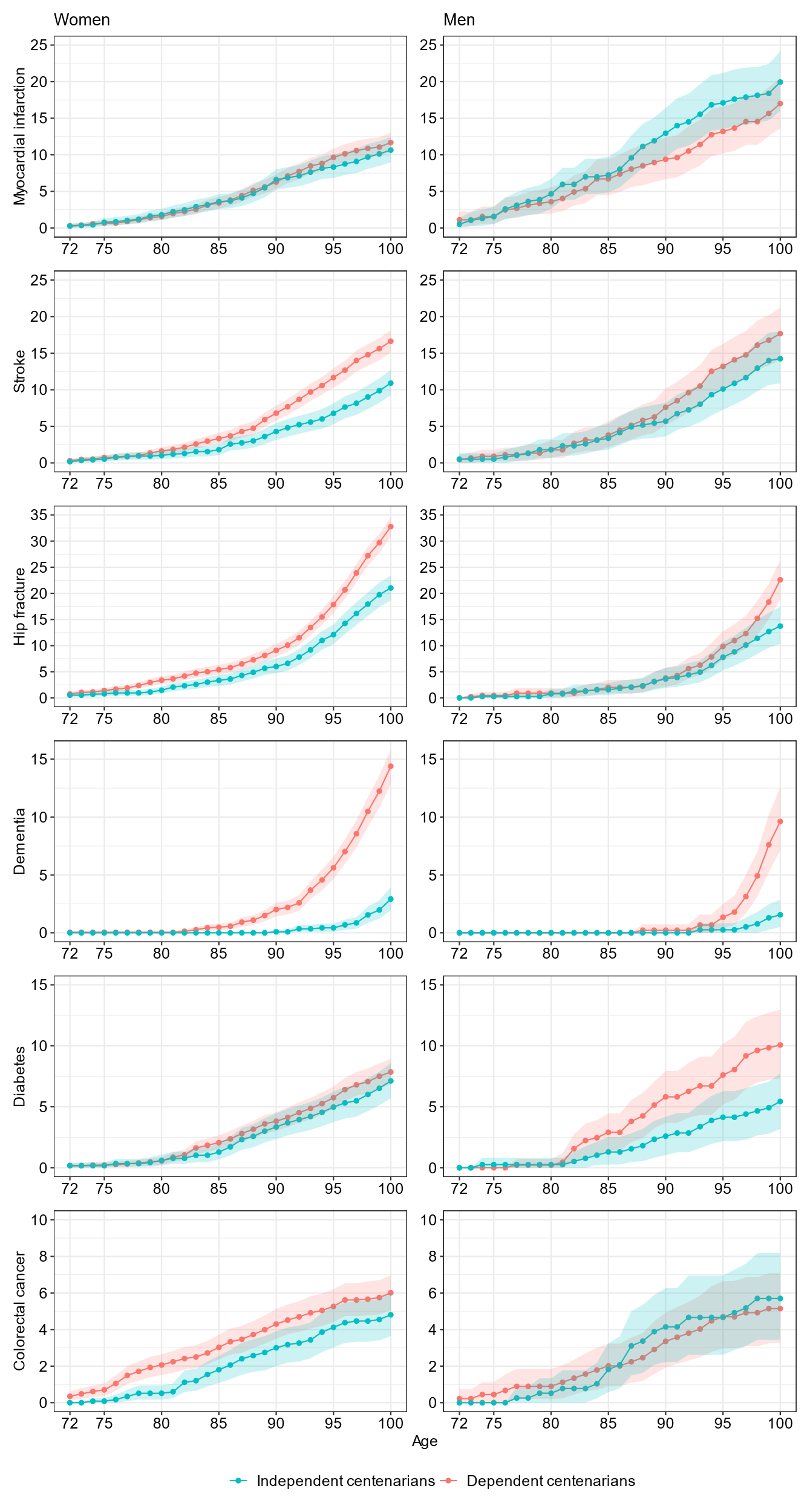
Supplementary Figure 4. Sex-stratified cumulative incidence of different diseases from the age of 72 and onwards for independent and dependent centenarians, respectively.**

Note. Shaded area represents the 95% confidence interval.
